# Baseline characteristics and comparability of older multimorbid patients with polypharmacy and general practitioners participating in a randomized controlled primary care trial

**DOI:** 10.1186/s12875-021-01488-8

**Published:** 2021-06-22

**Authors:** Katharina Tabea Jungo, Rahel Meier, Fabio Valeri, Nathalie Schwab, Claudio Schneider, Emily Reeve, Marco Spruit, Matthias Schwenkglenks, Nicolas Rodondi, Sven Streit

**Affiliations:** 1 Institute of Primary Health Care (BIHAM), University of Bern, Mittelstrasse 43, 3012 Bern, Switzerland; 2 Graduate School for Health Sciences, University of Bern, Bern, Switzerland; 3 Institute of Primary Care, University and University Hospital of Zurich, Zurich, Switzerland; 4 Department of General Internal Medicine, Inselspital, Bern University Hospital, University of Bern, Bern, Switzerland; 5 Quality Use of Medicines and Pharmacy Research Centre, UniSA: Clinical and Health Sciences, University of South Australia, Adelaide, South Australia Australia; 6 Geriatric Medicine Research, Faculty of Medicine and College of Pharmacy, Dalhousie University and Nova Scotia Health Authority, Halifax, NS Canada; 7 Department of Information and Computing Sciences, Utrecht University, Utrecht, The Netherlands; 8 Public Health & Primary Care, Leiden University Medical Centre, Leiden University, Leiden, The Netherlands; 9 Institute of Pharmaceutical Medicine (ECPM), University of Basel, Basel, Switzerland; 10 Epidemiology, Biostatistics and Prevention Institute, University of Zurich, Zurich, Switzerland

**Keywords:** Multimorbidity, Polypharmacy, Older adults, General practitioners, Clinical trial, External validity, Baseline characteristics

## Abstract

**Objectives:**

Recruiting general practitioners (GPs) and their multimorbid older patients for trials is challenging for multiple reasons (e.g., high workload, limited mobility). The comparability of study participants is important for interpreting study findings. This manuscript describes the baseline characteristics of GPs and patients participating in the ‘Optimizing PharmacoTherapy in older multimorbid adults In primary CAre’ (OPTICA) trial, a study of optimization of pharmacotherapy for multimorbid older adults. The overall aim of this study was to determine if the GPs and patients participating in the OPTICA trial are comparable to the real-world population in Swiss primary care.

**Design:**

Analysis of baseline data from GPs and patients in the OPTICA trial and a reference cohort from the FIRE (‘Family medicine ICPC Research using Electronic medical records’) project.

**Setting:**

Primary care, Switzerland.

**Participants:**

Three hundred twenty-three multimorbid (≥ 3 chronic conditions) patients with polypharmacy (≥ 5 regular medications) aged ≥ 65 years and 43 GPs recruited for the OPTICA trial were compared to 22,907 older multimorbid patients with polypharmacy and 227 GPs from the FIRE database.

**Methods:**

We compared the characteristics of GPs and patients participating in the OPTICA trial with other GPs and other older multimorbid adults with polypharmacy in the FIRE database. We described the baseline willingness to have medications deprescribed of the patients participating in the OPTICA trial using the revised Patients’ Attitudes Towards Deprescribing (rPATD) questionnaire.

**Results:**

The GPs in the FIRE project and OPTICA were similar in terms of sociodemographic characteristics and their work as a GP (e.g. aged in their fifties, ≥ 10 years of experience, ≥ 60% are self-employed, ≥ 80% work in a group practice). The median age of patients in the OPTICA trial was 77 years and 45% of trial participants were women. Patients participating in the OPTICA trial and patients in the FIRE database were comparable in terms of age, certain clinical characteristics (e.g. systolic blood pressure, body mass index) and health services use (e.g. selected lab and vital data measurements). More than 80% of older multimorbid patients reported to be willing to stop ≥ 1 of their medications if their doctor said that this would be possible.

**Conclusion:**

The characteristics of patients and GPs recruited into the OPTICA trial are relatively comparable to characteristics of a real-world Swiss population, which indicates that recruiting a generalizable patient sample is possible in the primary care setting. Multimorbid patients in the OPTICA trial reported a high willingness to have medications deprescribed.

**Trial registration:**

Clinicaltrials.gov (NCT03724539), KOFAM (Swiss national portal) (SNCTP000003060), Universal Trial Number (U1111-1226-8013)

**Supplementary Information:**

The online version contains supplementary material available at 10.1186/s12875-021-01488-8.

## Introduction

Globally, the population group of adults aged ≥ 65 years is growing faster than all other age groups combined. In 2019 one in every 11 persons was 65 years and over, this has been predicted to increase to one in six persons by the year 2050 [1]. With ageing societies, also come growing numbers of older adults with multiple chronic conditions. Multimorbid patients often use multiple medications and with polypharmacy comes a higher risk of using potentially inappropriate medications (PIMs). PIMs are medications for which the risk of potential adverse events outweighs the clinical benefits, such as when there are more effective and safer alternatives available for use in older adults [2]. The use of PIMs is associated with increased risk of adverse drug events, falls and cognitive impairment [3–6]. Patients with multimorbidity and polypharmacy often have complex healthcare needs, which in turn lead to substantial health services use and associated costs [7]. The use of potentially inappropriate medications is high in this patient group [8]. In this context, the ‘Optimizing PharmacoTherapy in older multimorbid adults In primary CAre’ (OPTICA) trial was launched with the aim of investigating whether an electronic clinical decision support tool can help GPs to optimise medication use of older multimorbid patients with polypharmacy.

Lack of external validity of clinical trials, the extent to which results can be generalised to the wider population, has been cited as a reason that interventions do not get adopted after publication of the study. One factor that can influence external validity is the characteristics of the participants recruited into the trial; that is, whether they are comparable (have similar characteristics) to those found in the real-world population [9].

Despite societal ageing and widespread multimorbidity, patients with chronic conditions and older adults in general are often underrepresented in clinical research [10, 11]. Evidence from studies of younger and healthier participants may not be generalizable to the broader older multimorbid population [12]. The reasons for the exclusion and general underrepresentation of complex older adults in research are multifaceted. On the one hand, studies often have inclusion and exclusion criteria to maximise participant retention and minimise variability among participants [13–15]. On the other hand, even if older multimorbid adults are not explicitly excluded, major barriers to recruiting this type of study participants include limited mobility (e.g. not being able to attend multiple appointments or complete certain tests), and in the case of cognitive impairment, inability to provide informed consent [14, 16, 17]. Additionally, the person identifying and selecting patients for recruitment (e.g. member of the research team or through healthcare professionals with established relationships) can impact the external validity of participants [18]. Use of routinely collected patient information to identify participants for clinical trials is a promising method to reduce the labour of recruitment. However, concerns exist about the error rate of using electronic medical records for this [19].

Not only can the recruitment of older multimorbid patients be challenging, so can the recruitment of GPs [20]. Previous studies found that time constraints, lack of training, fear of loss of professional autonomy as well as lack of rewards and recognition are barriers to research participation for physicians in general [21]. Conducting clinical research in the primary care setting comes with additional challenges. For instance, a lack of infrastructure, lack of financial remuneration of practice staff involvement, misunderstandings on how daily clinical work in general practice could accommodate the clinical research, and seasonal changes in workload [22, 23]. There is the concern that GPs with specific characteristics or attitudes can be motivated more easily to participate in clinical research. If true, it would mean that the results of an interventional study (such as our OPTICA trial) would not be generalizable to even the local context outside of those who participated in the trial. Overall, little is known about whether it is possible to recruit an externally comparable sample of older multimorbid patients and GPs for research in primary care.

Further, past medication optimization interventions in patients with polypharmacy have shown limited effect in changing medication use [24, 25] and/or clinical outcomes (e.g. mortality, cognitive decline) [26]. This may be due to patient resistance to medication changes and their unawareness of potentially inappropriate medication use [27]. It is therefore important to consider not only the characteristics of participants, but their attitudes as well.

The ‘Family medicine ICPC Research using Electronic medical records’ (FIRE) database is the largest Swiss electronic database containing anonymized routine patient data from the electronic medical records in > 10% of Swiss primary care practices. It also contains information about the GPs who regularly export data from their electronic medical records. The FIRE database therefore provides a unique opportunity to examine the likely external validity of the OPTICA study results to the wider Swiss general population in primary care.

The overall aim of this study was to determine if the GPs and patients participating in the OPTICA trial are comparable to the real-world population in Swiss primary care. We hypothesised that our broad inclusion criteria and support provided to participating GPs would result in recruitment of comparable participants. This information is not only important for interpreting the forthcoming results of the OPTICA trial (i.e. the likely external validity of the study findings), but can also inform the ability to recruit complex older adults for clinical trials in primary care.

Specifically, the aims of this manuscript were to:

1. Describe the baseline characteristics of participants (GPs and older patients with multimorbidity and polypharmacy) recruited to the OPTICA trial.
2. Compare the characteristics of GPs and patients participating in the OPTICA trial with those in the FIRE database.
3. Compare the characteristics of the patients recruited for OPTICA from random screening lists generated from electronic medical records with patients recruited through GP identification of eligible patients.
4. Describe the patients’ willingness to have medications deprescribed.

## Methods

### Study design and setting

For this analysis we used baseline data from the ongoing cluster-randomized controlled trial (cRCT) ‘Optimizing PharmacoTherapy in older multimorbid patients In primary CAre’ (OPTICA). We were able to compate the OPTICA study participants to reference cohorts from the ‘Family medicine ICPC Research using Electronic medical records’ (FIRE) project database, as all GPs who participated in the OPTICA trial regularly export data to the FIRE project. Details about these two research projects have been reported elsewhere [28, 29].

The FIRE project is the largest Swiss database collecting anonymized routine patient data from the electronic medical records in primary care practices since 2009 [28]. The following information is available in the FIRE database: administrative information (patient, age, and sex), diagnosis codes, laboratory and vital signs measurements, and prescribing information. As of October 2020, the database of the FIRE project contains data from the electronic medical records of more than 680 GPs (about 11% of all GPs in Switzerland [30]) and more than 830,000 patients (about 10% of the Swiss population) [31]. All GPs in Switzerland are invited to join the FIRE project if they use an electronic health record (EHR) program that is compatible with exporting anonymized data to the FIRE project.

The OPTICA trial is a cluster-randomized controlled trial, being conducted in primary care in the German speaking part of Switzerland. The aim of the OPTICA trial is to investigate whether the use of an electronic clinical decision support system, namely the ‘Systematic Tool to Reduce Inappropriate Prescribing’ (STRIP) Assistant [32], improves medication appropriateness compared to a standard care sham intervention in older multimorbid patients with polypharmacy. The STRIP Assistant (STRIPA) is based on the algorithms of the ‘Screening Tool to Alert doctors to Right Treatment’ (START) and ‘Screening Tool of Older Person’s Prescriptions’ (STOPP) version 2 [33], which are lists of medications generally considered to be inappropriate and appropriate in older adults, respectively [34]. The standard care sham intervention in the control group consists of a medication discussion between GPs and patients in accordance with usual care. The co-primary outcomes of the OPTICA trial are the ‘Medication Appropriateness Index’ (MAI) and the ‘Assessment of underutilization’ (AOU) [35–37]. More detailed background information about the OPTICA trial, the study intervention, and the FIRE project is reported in eAppendix 1 in the supplement.

### Participants

#### OPTICA trial

We present the inclusion and exclusion criteria for GPs and patients in the OPTICA trial in Table 1. To maximise the generalizability of the study population, we kept the exclusion criteria to a minimum. Patients were recruited through their GPs. GPs were instructed to use a random screening list generated from the data they exported to the FIRE project, but also had the flexibility to recruit other eligible patients after exhausting the screening lists. The calculated sample size of the OPTICA trial was 320 patients (details reported in the OPTICA protocol paper [29]).

**Table 1:** Inclusion and exclusion criteria for general practitioners and patients in the OPTICA trial^a^

**General practitioners**		**Patients**	
Inclusion criteria	Exclusion criteria	Inclusion criteria	Exclusion criteria
- Be a practicing GP in Switzerland - Complete online GCP training - Work with electronic medical records that are compatible with FIRE project^b^	- Non participation in the FIRE project -Another GP from the same practice already participating in the trial	- Be a patient of one of the participating GPs - Regularly see his/her GP, who is their main prescriber - ≥ 65 years or older - ≥ 3 chronic conditions - ≥ 5 chronic medications	-Participation in another clinical trial -Written informed consent not obtained from patient or from relative in case of cognitive impairment of the patient

*Abbreviations*: *GCP* Good Clinical Practice, *FIRE* Family medicine ICPC Research using Electronic medical records, *OPTICA* Optimising PharmacoTherapy In the multimorbid elderly in primary CAre, *GP* General practitioner

^a^As specified in: Jungo KT, Rozsnyai Z, Mantelli S, et al. ‘Optimising PharmacoTherapy In the multimorbid elderly in primary CAre’ (OPTICA) to improve medication appropriateness: study protocol of a cluster randomised controlled trial. BMJ Open 2019;9:e031080. https://doi.org/10.1136/bmjopen-2019-031080

^b^The FIRE project is a Swiss database with anonymized data from electronic health records of participating GPs. For the purpose of the OPTICA trial, we collect some relevant information for the trial through the FIRE project database, which is why the participation to the FIRE project has to be possible throughout the trial

#### FIRE project reference cohort

As of May 2019, around 520 GPs participated in the FIRE project. To define the target population of patients, we identified patients in the FIRE database who were at least 65 years and were prescribed at least 5 different medications at the time point of May 1^st^, 2019. The selection of reference GPs for the analyses took place as follows: GPs participating in the FIRE project, who were the GP of one of the patients included in the patient reference population (as described above) were included in the GP reference cohort (*n* = 227). GPs who participated in FIRE, but did not have any older multimorbid patients with polypharmacy (e.g. because they had only recently joined the project and did not yet export data) and those who took place in the OPTICA trial (*n* = 43) were excluded from the GP reference cohort. eFigure 1 in the supplement visualizes the creation of the reference cohorts.

### Data query and variables

From the FIRE database we extracted patients and GP characteristics. For GPs we extracted sociodemographic information and variables describing their work as GP (as shown in Table 2). For patients we extracted sociodemographic information, clinical parameters and variables describing their health services use (Table 3). All variables measuring health services use or reporting vital data and lab values were reported for the period of the last 12 months before May 2019.

**Table 2:** Baseline characteristics of general practitioners in the OPTICA trial compared to the general practitioners in the FIRE database

**Characteristics**	**OPTICA GPs (*N* = 43)**	**FIRE GPs (*N* = 227)**^**1**^	***P*-value**^**5**^	**Absolute standardized difference**^**6**^
Median age (IQR)	54 (45–60)	51 (44–58)	0.572	0.073
Median years since starting to work as GP (IQR)	15 (6–23)	10 (5–21)	0.302	0.159
Sex				
Women (%)	8 (19)	80 (35)	0.034	0.385
Men (%)	35 (81)	146 (65)		
Employment status				
Self-employed (%)	28 (70)	131 (63)	0.474	0.143
Employed (%)	12 (30)	76 (37)		
GP practice type				
Group practice (%)	36 (84)	200 (88)	0.452	0.126
Single practice (%)	7 (16)	27 (12)		
Location				
Non-urban (%)	17 (40)	51 (23)	0.022	0.375
Urban (%)	26 (60)	176 (78)		
Self-dispensation of medications in GP office^2^				
Yes (%)	25 (60)	175 (77)	0.046	0.386
No (%)	13 (31)	41 (18)		
Limited^3^ (%)	4 (10)	11 (5)		
Median work percentage (IQR)	80 (80–100)	80 (60–100)	0.020	0.401
Participation in integrated care model				
Yes	39 (93)	202 (95)	0.456	0.103
No	3 (7)	10 (5)		
Median percentage of eligible patients based on OPTICA inclusion criteria (IQR)^4^	6 (3–14)	7 (4–11)	0.614	0.287

*Abbreviations*: *GP* General practitioner, *IQR* Interquartile range, *OPTICA* Optimizing PharmacoTherapy in older multimorbid adults In primary CAre, *FIRE* Family medicine ICPC Research using Electronic medical records

^1^As of spring May 2019, excludes GPs who were part of the OPTICA trial and who did not have any eligible patients for the OPTICA trial

^2^Depending on the area/region they work in, GPs in Switzerland may be able to sell and dispense medications to their patients

^3^Only for selected medications

^4^ ≥ 5 medications from different ATC groups and age ≥ 65 years. The other inclusion and exclusion criteria were not implemented, as they had to be double checked by the GPs

^5^For categorical variables we performed a Fisher’s exact text and for continuous variables a Kruskal–Wallis test was performed; *P*-values of < 0.05 represent that there is evidence for a statistically significant difference between the two groups

^6^An imbalance between the two groups was previously defined as an absolute standardize difference value > 0.2

**Table 3:** Baseline characteristics of patients in the OPTICA trial compared to other multimorbid patients with polypharmacy in the FIRE database

**Characteristics**	**OPTICA study participants**^**1**^** (*N* = 323)**	**Patients in the FIRE database**^**2**^** (*N* = 22′907)**	***P*-value**^**3**^	**Absolute standardized difference**^**4**^
Median age (IQR)	77 (73–83)	78 (72–84)	0.630	0.053
Sex				
Women (%)	146 (45)	12′699 (55)	0.001	0.206
Men (%)	177 (55)	10′207 (45)		
Median number of chronic conditions (IQR)^5^	4 (3–6)	3 (3–5)	< 0.001	0.422
Median number of medications in the last 12 months (IQR)^6^	6 (5–9)	7 (5–8)	< 0.001	0.23
*Health services use (in the last 12 months)*				
Median number of consultations (IQR)	16 (10–25)	13 (7–22)	< 0.001	0.216
Median number of blood pressure measurements (IQR)	3 (2–5)	2 (1–4)	< 0.001	0.276
Median number of Body Mass Index measurements (IQR)	2 (1–3)	1 (1–3)	0.501	0.03
Median number of HbA1c measurements (IQR)	2 (1–4)	2 (1–3)	0.001	0.24
Median number of glomerular filtration rate (GFR) measurements (IQR)	2 (1–3)	1 (1–3)	< 0.001	0.208
Median number of lipid profile measurements (IQR)	1 (1–2)	1 (1–1)	0.166	0.093
*Lab values & vital signs (in the last 12 months)*				
Median systolic blood pressure (IQR)	138 (126–148)	138 (127–149)	0.541	0.023
Median diastolic blood pressure (IQR)	76 (70–83)	79 (72–85)	0.005	0.154
Median Body Mass Index (IQR)	29 (25–32)	28 (24–31)	0.235	0.101
Median HbA1c (IQR)	6.3 (5.7–7)	6.1 (5.6–6.9)	0.023	0.1
Median GFR (IQR)	66.2 (51.4–79.7)	68.3 (52.3–82.5)	0.314	0.041

*Abbreviations*: *BMI* Body Mass Index, *IQR* Interquartile range, *GFR* Glomerular filtration rate, *HbA1c* Hemoglobin A1C, *OPTICA* Optimizing PharmacoTherapy in older multimorbid adults In primary CAre, *FIRE* Family medicine ICPC Research using Electronic medical records

^1^Patients who participated in the OPTICA trial

^2^Patients eligible to participate in the OPTICA trial based on the inclusion and exclusion criteria, excludes patients who participated in the OPTICA trial

^3^For categorical variables we performed a Fisher’s exact text and for continuous variables a Kruskal–Wallis test was performed. *P*-values of < 0.05 represent that there is evidence for a statistically significant difference between the two groups

^4^An imbalance between the two groups was previously defined as an absolute standardize difference value > 0.2

^5^Chronic conditions were defined according to Lamers et al. and O’Halloran et al. [38, 39]

^6^Number of medications belonging to different groups defined by the Anatomical Therapeutic Chemical (ATC) classification system

The information on patients’ willingness to have medications deprescribed was collected in the baseline phone call conducted with participants in the OPTICA trial using the German translation of the revised Patients’ Attitudes Towards Deprescribing (rPATD) questionnaire. The original questionnaire was developed by Reeve et al. [38, 40]. The German translation was validated and used in a Swiss study on patients’ attitudes towards having medication deprescribed [39]. The rPATD questionnaire for both caregivers and patients contains two global questions as well as questions grouped into four factors: medication burden, mediation appropriateness, concerns about stopping, and involvement. There are four to five questions per factor, which can be used to calculate a factor score. Each factor score ranges from 1 to 5 [40].

### Statistical analysis

First, we compared the characteristics of GPs participating in the OPTICA trial with those of the reference GPs in the FIRE database. Second, we compared the characteristics of the OPTICA study participants with those of other older, multimorbid patients in the FIRE database. Third, we compared the characteristics of the patients recruited for OPTICA from the random screening lists with the OPTICA patients recruited directly by GP identification of eligible participants (i.e. not from the screening lists). Finally, we described patients’ willingness to have medications deprescribed. We also performed a sensitivity analysis, by comparing the characteristics of the OPTICA study participants with all other older patients of the same GP only.

Categorical data are presented as frequencies and percentages, and continuous variables as median and interquartile range (IQR), as the variables were non-normally distributed. For categorical variables we performed a Fisher’s exact text and for continuous variables a Kruskal–Wallis test was performed, as defined in the R package “tableone” [41]. For this study, if the *p*-value was < 0.05 we concluded that there was sufficient evidence to say that the groups were statistically different. We also calculated standardized differences, which can be used to compare balances in measured variables [42]. While *p*-values were used for the statistical hypothesis testing, absolute standardized difference (ASD) values helped quantify the differences between groups. An ASD value > 0.2 has previously been defined as representing an imbalance between two groups [43]. Hence for the purpose of this study we considered a maximum threshold of 0.2 for ASD value as being acceptable in terms of comparability of the two groups. The group comparisons were performed using the statistical software package R (Version 3.6.3) [44].

The analyses on patients’ willingness to deprescribe were performed using the statistical software Stata 15.1 (StataCorp, College Station, TX, USA). We calculated the four factor scores (involvement, burden, appropriateness, and concerns about stopping) as described previously [40]. Each score is calculated based on responses to the 5 items within each factor of the rPATD questionnaire and ranges from 1–5. In addition, we present the responses to the two stand-alone statements from the rPATD (“Overall, I am satisfied with my current medicines” and “If my doctor said it was possible I would be willing to stop one or more of my regular medicines”).

### Patient and public involvement

As described in the OPTICA protocol paper [29], GPs and older patients with multimorbidity and polypharmacy are represented in the independent Safety and Data Monitoring Board of the OPTICA trial. GPs participating in the OPTICA trial receive regular newsletters. At the end of the study, study participants are informed about their study allocation and the results of the study.

## Results

**Fig. 1:**
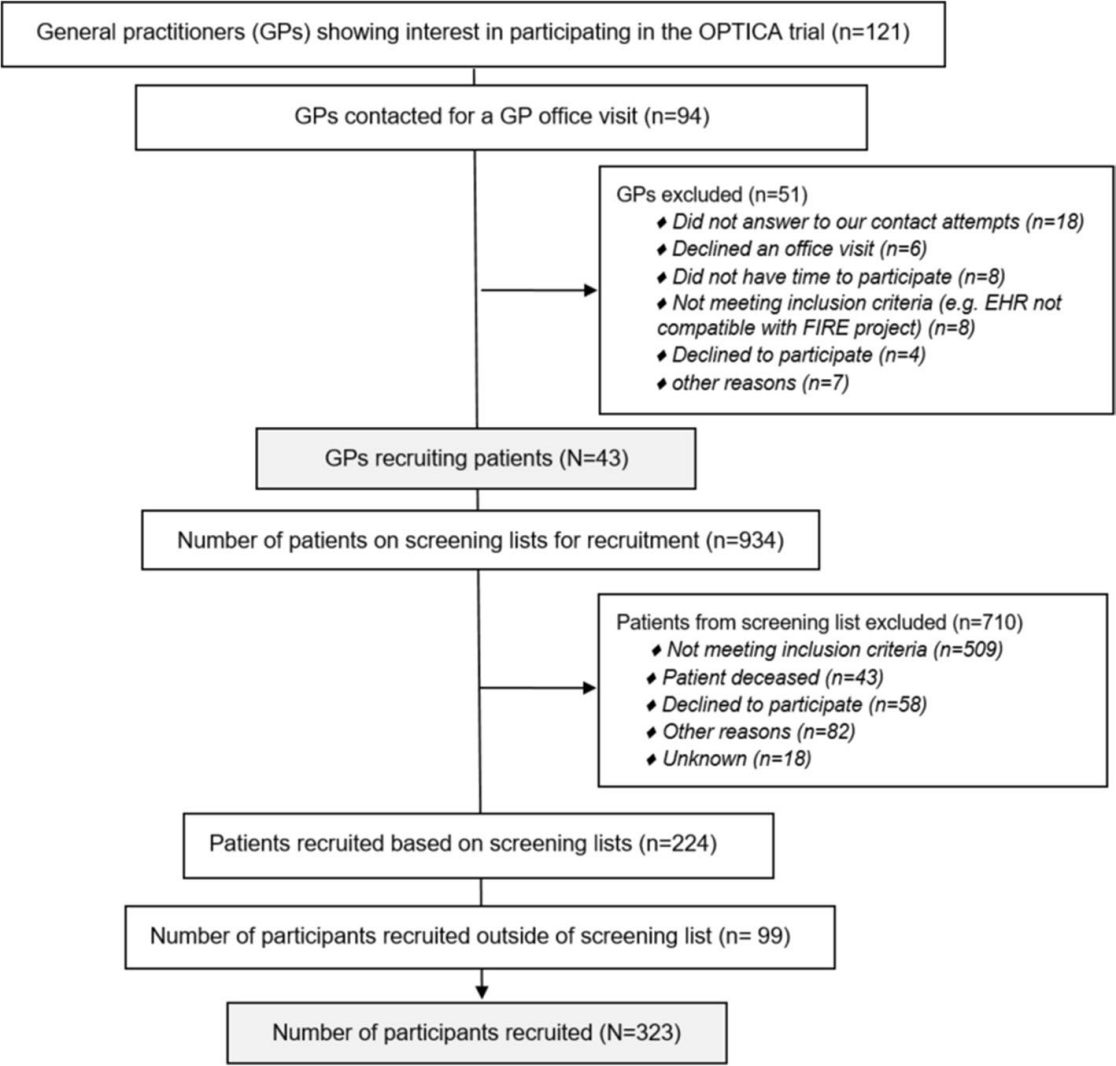
Flow chart of recruitment of general practitioners and patients in the OPTICA trial^1^. ^1^cluster-randomized controlled trial in Swiss primary care. Abbreviations: OPTICA = Optimizing PharmacoTherapy in older multimorbid adults In primary CAre

The process of the recruitment of GPs and patients in the OPTICA trial is shown in the trial flow chart (Fig. 1). Out of 121 GPs showing interest in the OPTICA trial, 94 were contacted for a recruitment visit in their GP office (explanation of study design, tasks for participating GPs, and if needed, installation of FIRE data export tools), and 43 were recruited. Out of 934 patients on the screening lists, 224 were recruited. Additionally, 99 patients (30.6% of the total patients recruited) were recruited through GP identification of eligible patients (outside of the screening list).

### What types of GPs participated in the OPTICA trial, and how did they compare to the non-participating GPs from FIRE?

As shown in Table 2, GPs who participated in OPTICA and those from the FIRE reference cohort were in their fifties on average (OPTICA median = 54, FIRE median = 51), had several years of experience working as a GPs (OPTICA median = 15, FIRE median = 10), and the majority were self-employed (OPTICA self-employed = 70%, FIRE self-employed = 63%). The GPs in the FIRE reference cohort and OPTICA were similar in terms of age, median years since starting to work as a GP, employment status, GP practice type, and participation in integrated care models (*p*-values > 0.05 and absolute standardized differences (ASD) < 0.2). We found differences between OPTICA and GPs from the FIRE reference cohort with regards to sex (lower proportion of female GPs in the OPTICA trial), location (greater proportion of OPTICA GPs in non-urban areas), and self-dispensing of medications in GP office (lower proportion of OPTICA GPs than FIRE GPs). The median work percentage was 80% in both groups (4-day week), but *p*-value and ASD showed that the distribution of the work percentages was different between groups.

### What types of patients consented to participate in the OPTICA trial, and how did they compare to non-participants?

As shown in Table 3, patients participating in the OPTICA trial were relatively comparable to other older patients with multimorbidity in the FIRE reference cohort with regards to their clinical characteristics and health services use. On average, patients were in their late seventies (OPTICA median = 77, FIRE median = 78), and regularly saw their GP (OPTICA median consultation counts in the last 6 months = 16, FIRE median = 13). We did not find evidence for a difference between the groups with regards to age, the median number of Body Mass Index (BMI) measurements, the median number of lipid profile measurements, median systolic blood pressure, median BMI and median number of glomerular filtration rate (GFR) measurements (*p*-values > 0.05 and ASD < 0.1 for all these variables). Median diastolic blood pressure and median HbA1c values were found to be statistically significant between groups, but the ASD was close or equal to 0.1. For most of the remaining variables, we found statistically significant differences and standardized differences of around 20% (e.g. sex, median number of consultations, median number of medications, etc.). On average, patients in the OPTICA trial had more chronic conditions (OPTICA median = 4, FIRE median = 3, ASD = 0.422), but less medications (OPTICA median = 6, FIRE median = 7, ASD = 0.23). Within patients of the same GP, patients participating in OPTICA were comparable to patients not participating in OPTICA (eTable 1 in the supplement).

### How did patients recruited from random screening lists and other patients compare?

Two hundred and twenty-four patients were recruited from the random screening lists and 99 patients were recruited outside of these lists. The comparison of these two group (Table 4) found that they were comparable. We only found a statistically significant difference concerning median number of consultations (*p* = 0.031) and number of BMI measurements (*p* = 0.022).

**Table 4:** Baseline characteristics of patients in the OPTICA trial who were recruited from the screening list and those who were recruited outside of the screening list

**Characteristics**	**OPTICA study participants from screening list (*N* = 224)**	**OPTICA study participants not from screening list (*N* = 99)**	***P*-value**^**1**^	**Absolute standardized difference**^**2**^
Median age (IQR)	77 (72–82)	79 (74–84)	0.088	0.183
Sex				
Women (%)	106 (47)	40 (40)	0.276	0.14
Men (%)	118 (53)	59 (60)		
Median number of chronic conditions (IQR)	4 (3–6)	4 (3–6)	0.774	0.086
Median number of medications in the last 12 months (IQR)	6 (5–9)	7 (3–9)	0.464	0.16
*Health services use (in the last 12 months)*				
Median number of consultations (IQR)	17 (10–26)	14 (9–21)	0.031	0.303
Median number of blood pressure measurements (IQR)	3 (2–6)	3 (1–5)	0.197	0.034
Median number of Body Mass Index measurements (IQR)	1 (1–2)	2 (1–3)	0.255	0.329
Median number of HbA1c measurements (IQR)	2 (1–3)	2 (1–4)	0.332	0.147
Median number of glomerular filtration rate (GFR) measurements (IQR)	2 (1–3)	2 (1–3)	0.901	0.045
Median number of lipid profile measurements (IQR)	1 (1–2)	1 (1–2)	0.667	0.101
*Lab values & vital signs (in the last 12 months)*				
Median systolic blood pressure (IQR)	137 (125–147)	139 (130–150)	0.397	0.102
Median diastolic blood pressure (IQR)	76 (70–83)	76 (71–83)	0.801	0.078
Median Body Mass Index (IQR)	29 (25–32)	29 (25–33)	0.902	0.015
Median HbA1c (IQR)	6.3 (5.8–7.0)	6.4 (5.6–7.0)	0.991	0.02
Median GFR (IQR)	66.5 (53.4–80.1)	62.7 (48–6-78.9)	0.264	0.167

*Abbreviations*: *BMI* Body Mass Index, *IQR* Interquartile range, *GFR* Glomerular filtration rate, *HbA1c* Hemoglobin A1C, *OPTICA* Optimizing PharmacoTherapy in older multimorbid adults In primary CAre

^1^For categorical variables we performed a Fisher’s exact text and for continuous variables a Kruskal–Wallis test was performed. *P*-values of < 0.05 represent that there is evidence for a statistically significant difference between the two groups

^2^An imbalance between the two groups was previously defined as an absolute standardize difference value > 0.2

### What was study participants’ willingness to have medications deprescribed?

As shown in Table 5, at baseline of the OPTICA trial, the majority of patients in the OPTICA trial (> 90%) reported to be satisfied with their current medications. Furthermore, most of the study participants (> 80%) reported to be willing to stop one or more of their medications if their doctor said that it was possible. The OPTICA study participants reported to be involved in their medication use (median involvement score = 4.8 (IQR = 4.2–5.0); score can range from 1 to 5, with 5 representing a high reported involvement). The median medication burden score was 2.2 (IQR = 1.6–2.8) and the concerns about stopping score was 1.6 (IQR = 1.0–2.4). Results of caregivers who completed the caregiver rPATD (where the patient was unable to complete the questionnaire due to cognitive impairment, *n* = 16) are shown in Table 5.

**Table 5:** Patients’ and caregivers’ willingness to have medications deprescribed assessed with ‘revised Patients’ Attitudes Towards Deprescribing’ (rPATD) questionnaire^a^

	**OPTICA patients (*n* = 298)**	**Caregivers of OPTICA participants with cognitive impairment (*n* = 16)**
“Overall, I am satisfied with my current medicines” (%) and respectively “Overall, I am satisfied with my care recipient’s current medicines”		
Strongly agree	215 (72.2)	11 (68.7)
Agree	64 (21.5)	5 (31.3)
Unsure	4 (1.3)	-
Disagree	11 (3.7)	-
Strongly disagree	4 (1.3)	-
“If my doctor said it was possible I would be willing to stop one or more of my regular medicines” (%) and respectively “If their doctor said it was possible I would be willing to stop one or more of my care recipient’s medicines”		
Strongly agree	224 (75.2)	10 (62.5)
Agree	38 (12.8)	3 (18.8)
Unsure	9 (3.0)	1 (6.3)
Disagree	14 (4.7)	1 (6.3)
Strongly disagree	13 (4.4)	1 (6.3)
**Factor scores**		
**Involvement**: Median involvement in medication management score (IQR)	4.8 (4.2–5.0)	4 (3.4–5.0)
[range: 1–5, the higher the score the more ‘involved’ patients are with their medications and caregivers with the medications of the person they care for]		
**Burden**: Median perceived burden of medications score (IQR)	2.2 (1.6–2.8)	2.3 (1.3–3.8)
[range: 1–5, the higher the score the more burdensome patients and caregivers perceive/view/experience the medications to be]		
**Appropriateness**: Median belief in appropriateness of medications score (IQR)	3.8 (3.4–4.2)	3.8 (3.4–4.2)
[range: 1–5, the higher the score the more appropriate patients respectively caregivers perceive/view/experience the medications]		
**Concerns about stopping**: Median concerns about stopping medications score (IQR)	1.6 (1.0–2.4)	1.2 (0.8–1.6)
[range: 1–5, the higher the score the potential concerns patients respective caregivers have about stopping one or more of the medications]		

*Abbreviations*: *OPTICA* Optimizing PharmacoTherapy in older multimorbid adults In primary CAre, *rPATD* Revised Patients’ Attitudes Towards Deprescribing

^a^Reeve, E., Low, L. F., Shakib, S., & Hilmer, S. N. (2016). Development and Validation of the Revised Patients’ Attitudes Towards Deprescribing (rPATD) Questionnaire: Versions for Older Adults and Caregivers. Drugs & Aging, 33(12), 913–928. Since the scores were not normally distributed we decided to present the medians

## Discussion

To inform the likely external validity of the results of the OPTICA trial, we compared the characteristics of our participating GPs and patients to a Swiss real-world reference cohort. We also examined the characteristics of patients recruited based on random screening lists (created from electronic medical records) and those recruited outside of these lists by their GP to see whether a bias in the selection may exist. Finally we explored the willingness of patients in OPTICA to have medications deprescribed which allows us to reflect on the possible impact that this may have on the outcomes of the trial and compare them to previously studied populations. From our analyses we have some confidence that the findings of the OPTICA study will be generalizable to the broad Swiss population of GPs and patients. We found that the GPs in the FIRE project and OPTICA were similar in terms of sociodemographic characteristics and their work as a GP (e.g. age, experience as GP, employment status, and GP practice type). We also found that patients participating in the OPTICA trial and patients in the FIRE database were comparable in terms of age, median number of certain lab and vital data measurements (e.g. BMI, lipid profile, GFR measurements) and certain clinical characteristics (e.g. systolic blood pressure, BMI). For the variables that differed between the two groups according to the statistical tests, the absolute standardized differences were generally around 0.2 (or 20%), with an imbalance of the two groups having previously been defined as > 0.2. Patients who participated in the OPTICA trial reported a high level of willingness to stop one or more of their medications.

Overall, our study results showed that GPs who participated in the OPTICA trial and those who participated in the FIRE project were comparable in most of the variables examined. Previous research showed that high performing physicians are more likely to participate in research [45]. When looking at the patient data, we observed that OPTICA patients had more chronic conditions, but less medications. The absolute standardized differences indicate some imbalances between the groups on these variables. While one can argue about whether the differences are clinically relevant, this observation could indicate that GPs in the OPTICA trial may have been more proactive in reviewing the medications of their patients than other GPs. If the latter was the case, this would mean that the intervention of the OPTICA trial may be limited in its effect (i.e. if the patients had little room for further optimisation of their medications). We also found differences in sex, location and self-dispensing between GPs in both groups. These differences may have stemmed from the recruitment strategy used in the OPTICA trial, which in the context of difficulties of recruiting GPs for clinical research focused (and therefore needing to optimise GP recruitment) did not specifically recruit based on their baseline characteristics. The sex composition of the OPTICA GPs could affect the final results, since female physicians have been found to be less likely to make deprescribing decisions [46].

We found that the multimorbid older patients who participated in the OPTICA trial were comparable to those in the FIRE database in terms of sociodemographic variables, health services use and clinical characteristics. For the variables were there was a statistically significant difference between the groups, most had standardized differences close but not passing the ASD threshold of 0.2 for meaningful differences between the groups (e.g. number of medications: OPTICA median = 6, FIRE median = 7, ASD = 0.23, number of consultations OPTICA median = 16, FIRE median = 13, ASD = 0.216). There is a lower proportion of female participants in the OPTICA trial than in the reference FIRE cohort. However, since no difference in willingness to deprescribe according to sex has been identified [39, 47], we do not anticipate that this sex imbalance will affect the results of the OPTICA trial.

We found that the trial participants recruited from the random screening lists (around two thirds of patients) and those who were recruited outside of these lists (around one third of patients) were comparable. While systematic differences in recruitment behaviour (i.e. differential recruitment [48]) has been reported previously in the context of a cluster-randomized controlled trial in primary care (UK BEAM trial) [49], we did not find evidence for a bias in the selection of participants in the OPTICA trial. The UK BEAM trial reported, for example, that patients in participating practices were experiencing milder back pain (which the intervention targeted) than those in the control group and thus highlighted the potential for the recruitment process to bias study results [49]. The use of random screening lists helped to standardize patient recruitment but, in light of the imperfect nature of the screening lists, we also allowed GPs to recruit patients who were not on these lists. We assumed that giving participating GPs some flexibility in the recruitment process would allow them to better integrate recruitment into their regular practice and would therefore optimise recruitment.

Concerning patients’ willingness to deprescribe, we found that the OPTICA study participants had a high involvement in their medication use and > 80% were willing to stop one or more of their regular medications if their doctor told them this was possible. These findings are in line with previous research. Another study conducted in Switzerland found that 77% of older adults would be willing to stop one or more of their medications [39] and similar proportions were found in studies in other countries (88% in Australia [47], 92% in the United States [50], 83% in Singapore [51]). While these numbers have to be interpreted with caution (e.g. social desirability bias, not medication specific, hypothetical nature of the question), it shows that older patients may be open to optimizing their medication use through deprescribing. We also found the factor scores to be comparable to the results from a study in Australian older adults [47]. This information is crucial for implementing medication optimization interventions, and in the context of the OPTICA trial, it shows that patients’ attitudes towards deprescribing may not be a barrier to implementation of deprescribing.

While the results presented in this manuscript are primarily Swiss-specific, we can draw a more broadly applicable conclusion; it appears to be possible to recruit a sample of study participants in primary care trials that is comparable to real life cohorts.

### Strengths & limitations

The OPTICA trial had a low number of exclusion criteria, which facilitated broad recruitment of study participants. However, the analyses in this manuscript have several limitations. First, in Switzerland there are no complete GP or patient registries. The FIRE project maintains the only primary care database in Switzerland of this size, but it does not include all GPs in Switzerland, and in turn, does not include all patients in Switzerland. In Switzerland, not all GPs use electronic health record programs. The use of electronic health records in Switzerland increased from around 40 to > 70% from 2012 to 2020 [17, 34], but remains lower than in other high-income countries. Furthermore, not all GPs who fulfil the eligibility criteria self-select to participate in the FIRE project. This raises the question of the representativeness of the GPs in the FIRE database. However, two recent assessments of the Swiss GP workforce showed that the GPs in the FIRE project are comparable to the entire GP workforce in terms of age, sex, experience as GP and work percentage (eTable 2 in the supplement). These similarities between OPTICA, FIRE and all GPs in Switzerland signify that the recruitment of an externally comparable sample of GPs is possible in randomized clinical trials in the Swiss primary care setting. This confirms previous evidence from the UK, which showed that achieving good levels of external validity was possible in clinical trials in primary care [52]. However, due to the lack of patient registries, we cannot comment on the comparability of patients in the FIRE project and Swiss patients in general. While the analyses presented in this manuscript do not confirm external validity of the forthcoming OPTICA trial results, they do facilitate future interpretation of our findings.

Next, inherent to routine medical databases, like the FIRE database, is a certain risk of information bias and missing data as information is only collected when it is clinically relevant [53]. Since we used data from before the OPTICA study intervention started, we assume that both our groups would have been affected by the same potential sources of bias. Despite the similarities found between the FIRE and Swiss GP workforce in terms of sociodemographic and work-related characteristics, we were unable to compare other important characteristics between the two groups (e.g. quality of care, relationship and trust between doctor and patient). Our finding that the patients included in the OPTICA trial had less medication but more chronic conditions than the reference cohort could reflect the selection of “good performers” which may bias the findings of the OPTICA trial. Our analysis of patients’ willingness to deprescribe was limited to patients in the OPTICA trial and could not be compared directly to the reference cohort and this questionnaire is not used in regular clinical care. Other limitations related to the rPATD are that it asks hypothetical questions, it is not specific to certain medications, and it might be subject to social desirability bias. Furthermore, for the purpose of the OPTICA trial the rPATD was translated from English to German; back-translation and piloting was conducted to increase the validity of the translation, but other measures of validation and reliability of the translation in the local context were not conducted (e.g. test–retest reliability). Finally, due to the uncertainties surrounding the absolute standardized differences, we decided to present both *p*-values and ASD. While there may be debate of the cut off to use for ASD, we used > 0.2 as this has been recommended by Yang et Dalton [42, 43]. If we considered a smaller threshold, such as > 0.1, it would not have changed our conclusions about the groups being comparable.

## Conclusion

In the OPTICA trial, it was possible to recruit GPs and their older patients with multimorbidity and polypharmacy that are generally comparable to a real-world reference cohort of GPs and older patients with multimorbidity and polypharmacy in Switzerland. The observed similarities between OPTICA, FIRE and all Swiss GPs signify that the recruitment of an externally valid sample of GPs is possible in randomized clinical trials in the Swiss primary care setting. The findings from this manuscript about the baseline characteristics of study participants will be crucial for interpreting the wider applicability of the OPTICA study intervention and its findings. Ensuring that clinical trials recruit comparable populations is crucial for improving the care of older multimorbid patients, which have previously been underrepresented in clinical research.

## Supplementary Information

**Additional file 1: eAppendix 1.** Information about the OPTICA trial and the FIRE project. **eTable 1.** Baseline characteristics of patients in the OPTICA trial compared to other multimorbid patients with polypharmacy in the FIRE database who also were patients of the general practitioners participating in the OPTICA trial. **eTable 2.** Baseline characteristics of Swiss general practitioners who participated in the Workforce-Study. **eFigure 1.** FIRE database, FIRE reference cohorts and OPTICA trial participants.

## Data Availability

The FIRE database can be accessed at any time by the scientific team of the institute. For external requests, access has to be requested from the head of the institute.

## Abbreviations

ASD: Absolute standardized difference
FIRE: ‘Family medicine ICPC Research using Electronic medical records’
GCP: Good Clinical Practice
GP: General practitioner
OPTICA: Optimising PharmacoTherapy In the multimorbid elderly in primary CAre
rPATD: Revised Patients’ Attitudes Towards Deprescribing
