# Supplementary material for: Baseline characteristics and comparability of older multimorbid patients with polypharmacy and general practitioners participating in a randomized controlled primary care trial
Source: BMC Fam Pract. 2021 Jun 22;22:123. doi: 10.1186/s12875-021-01488-8 (PMC8220761; doi:10.1186/s12875-021-01488-8)
Supplement: Supplementary file 1 — Additional file 1: eAppendix 1. Information about the OPTICA trial and the FIRE project. eTable 1. Baseline characteristics of patients in the OPTICA trial compared to other multimorbid patients with polypharmacy in the FIRE database who also were patients of the general practitioners participating in the OPTICA trial. eTable 2. Baseline characteristics of Swiss general practitioners who participated in the Workforce-Study. eFigure 1. FIRE database, FIRE reference cohorts and OPTICA trial participants. [file 12875_2021_1488_MOESM1_ESM.docx]

**Baseline characteristics and comparability of older multimorbid patients with polypharmacy and general practitioners participating in a randomized controlled primary care trial**

**Supplementary Material**

Katharina Tabea Jungo^1,2^, Rahel Meier^3^, Fabio Valeri^3^, Nathalie Schwab^1,4^, Claudio Schneider^4^, Emily Reeve^5,6^, Marco Spruit^7,8^, Matthias Schwenkglenks^9,10^, Nicolas Rodondi^1,4^, Sven Streit^1^

^1^ Institute of Primary Health Care (BIHAM), University of Bern, Bern, Switzerland.

^2^ Graduate School for Health Sciences, University of Bern, Switzerland

^3^ Institute of Primary Care, University of Zurich, Zurich, Switzerland

^4^ Department of General Internal Medicine, Inselspital, Bern University Hospital, University of Bern, Bern, Switzerland

^5^ Quality Use of Medicines and Pharmacy Research Centre, UniSA: Clinical and Health Sciences, University of South Australia, Adelaide, South Australia, Australia

^6^ Geriatric Medicine Research, Faculty of Medicine and College of Pharmacy, Dalhousie University and Nova Scotia Health Authority, Halifax, NS, Canada

^7^ Department of Information and Computing Sciences, Utrecht University, Utrecht, The Netherlands

^8^ Public Health & Primary Care, Leiden University Medical Centre, Leiden University, The Netherlands

^9^ Institute of Pharmaceutical Medicine (ECPM), University of Basel, Basel, Switzerland

^10^ Epidemiology, Biostatistics and Prevention Institute, University of Zurich, Zurich, Switzerland

**Table of content**

**eAppendix 1.** Information about the OPTICA trial and the FIRE project.

**eTable 1.** Baseline characteristics of patients in the OPTICA trial compared to other multimorbid patients with polypharmacy in the FIRE database who also were patients of the general practitioners participating in the OPTICA trial.

**eTable 2.** Baseline characteristics of Swiss general practitioners who participated in the Workforce-Study.

**eFigure 1**. FIRE database, FIRE reference cohorts and OPTICA trial participants.

**eAppendix 1.** Information about the OPTICA trial and the FIRE project.

***FIRE project***

The FIRE project is the largest Swiss database collecting anonymized routine patient data from the electronic medical records in primary care practices since 2009 [1]. The following information is available in the FIRE database: administrative information (patient, age, and sex), diagnosis codes, laboratory and vital signs measurements, and prescribing information. As of October 2020, the database of the FIRE project contains data from the electronic medical records of more than 680 GPs (about 11% of all Swiss GPs [2]) and more than 830’000 patients (about 10% of the general population) [3]. All Swiss GPs are invited to join the FIRE project if they use an electronic health record (EHR) program that is compatible with exporting anonymized data to the FIRE project. Six of the most commonly used EHR programs in the German speaking part of Switzerland are compatible with FIRE. GPs who participate in the FIRE project export selected, anonymized data from their EHR every two months. In return, the GPs receive feedback reports, which they can use for quality assurance purposes.

***OPTICA trial***

The protocol for the OPTICA trial is described elsewhere in detail [4]. Briefly, the OPTICA trial is a cluster randomized controlled trial, being conducted in primary care in the German speaking part of Switzerland. The aim of the OPTICA trial is to investigate whether the use of an electronic clinical decision support system, namely the ‘Systematic Tool to Reduce Inappropriate Prescribing’ (STRIP) Assistant [5], improves medication appropriateness compared to a standard care sham intervention in older multimorbid patients with polypharmacy. The STRIP Assistant (STRIPA) is based on the algorithms of the ‘Screening Tool to Alert doctors to Right Treatment’ (START) and ‘Screening Tool of Older Person’s Prescriptions’ (STOPP) version 2 [6] which are lists of medications generally considered to be inappropriate and appropriate in older adults, respectively [7]. The standard care sham intervention in the control group consists of a medication discussion between GPs and patients in accordance with usual care. The co-primary outcomes of the OPTICA trial are the ‘Medication Appropriateness Index’ (MAI) and the ‘Assessment of underutilization’ (AOU) [8-10]. Secondary outcomes include: degree of polypharmacy, degree of overprescribing, degree of underprescribing, number of falls and fractures, quality of life, the amount of formal and informal care received by patients, survival, patients’ quality adjusted life years (QALYs), patients’ medical costs, cost-effectiveness of the intervention, percentage of recommendations accepted and rejected by GPs, and patients’ willingness to have medications deprescribed. The latter is assessed using a validated questionnaire: the ‘revised Patient Attitudes Towards Deprescribing” questionnaire, which provides insights into patients' willingness for deprescribing and potential barriers to deprescribing [11,12]. Patients are followed-up for one year. At baseline, 6 months and 12 months, data for the OPTICA trial was collected by conducting phone calls (e.g. sociodemographic information, etc.) and from the FIRE database (e.g. medications, diagnoses, lab values and vital data).

In the OPTICA trial, we tried early on to establish recruitment and retention strategies designed to overcome challenges linked to GP recruitment (e.g. personal visits to explain the study, provide rapid response to questions and problems faced by participating GPs) [13].The recruitment of GPs for the OPTICA trial began in autumn 2018 and ended in late 2019. Clustering occurred on the level of the GP. The participating GPs each formed a cluster. The patient recruitment took place from December 2018 to February 2020 and was done directly by the participating GPs. To standardize the selection of eligible patients, GPs received screening lists with potentially eligible patients. These screening lists were created based on data previously exported to the FIRE database and included random sample of their own patients who were potentially eligible (based on age and polypharmacy). However, due to the nature of the routine data collected in the FIRE project, the provided screening list were not 100% accurate (e.g., inclusion of dead patients, patients who had changed their GP). Due to this, GPs were allowed to recruit other patients that they directly identified who fulfilled the inclusion and exclusion criteria.

**eTable 1. Baseline characteristics of patients in the OPTICA trial compared to other multimorbid patients with polypharmacy in the FIRE database who also were patients of the general practitioners participating in the OPTICA trial.**

| **Characteristics** | **OPTICA study participants**  **(N=323)** | **Other patients of same general practitioners**  **(N=3’549)** | **p-value^1^** | **Absolute standardized difference^2^** |
| --- | --- | --- | --- | --- |
|  |  |  |  |  |
| Median age (IQR) | 78 (72-85) | 77 (73-83) | 0.31 | 0.089 |
| Sex | | |  |  |
| Women (%) | 1858 (52) | 146 (45) | 0.015 | 0.143 |
| Men (%) | 1691 (48) | 177 (55) |  |  |
| Median number of chronic conditions (IQR) | 4 (3-5) | 4 (3-6) | <0.001 | 0.164 |
| Median number of medications in the last 12 months (IQR) | 7 (5-9) | 6 (5-9) | <0.001 | 0.268 |
| *Health services use (in the last 12 months)* | | | |  |
| Median number of consultations (IQR) | 14 (7-23) | 16 (10-25) | <0.001 | 0.173 |
| Median number of blood pressure measurements (IQR) | 2 (1-4) | 3 (2-5) | <0.001 | 0.293 |

| Median number of Body Mass Index measurements (IQR) | 1 (1-2) | 1.5 (1-3) | 0.186 | 0.069 |
| --- | --- | --- | --- | --- |

| Median number of HbA1c measurements (IQR) | 2 (1-3) | 2 (1-4) | 0.005 | 0.214 |
| --- | --- | --- | --- | --- |
| Median number of glomerular filtration rate (GFR) measurements (IQR) | 1 (1-2) | 2 (1-3) | <0.001 | 0.286 |

| Median number of lipid profile measurements (IQR) | 1 (1-1) | 1 (1-2) | 0.006 | 0.235 |
| --- | --- | --- | --- | --- |

| *Lab values & vital signs (in the last 12 months)* | | |  |  |
| --- | --- | --- | --- | --- |
| Median systolic blood pressure (IQR) | 138 (128-149) | 138 (126-148) | 0.533 | 0.008 |
| Median diastolic blood pressure (IQR) | 78 (71-84) | 76 (70-83) | 0.124 | 0.077 |
| Median Body Mass Index (IQR) | 28 (24-31) | 29 (25-32) | 0.146 | 0.146 |
| Median HbA1c (IQR) | 6.1 (5.6-6.9) | 6.3 (5.7-7.0) | 0.036 | 0.082 |
| Median GFR (IQR) | 69.0 (53.1-82.7) | 66.2 (51.4-79.7) | 0.223 | 0.052 |

| **^1^** For categorical variables we performed a Fisher’s exact text and for continuous variables a Kruskal-Wallis test was performed. **^2^** An imbalance between the two groups was previously defined as an absolute standardized difference value >0.2. Abbreviations: BMI=Body Mass Index; IQR= Interquartile range; GFR=Glomerular filtration rate; HbA1c=Hemoglobin A1C; OPTICA = Optimizing PharmacoTherapy in older multimorbid adults In primary CAre; FIRE = Family medicine ICPC Research using Electronic medical records |
| --- |

**eTable 2. Baseline characteristics of Swiss general practitioners who participated in the Workforce-Study.**

| **Characteristics** | **General practitioners**  **(N=2’037)** |
| --- | --- |
|  |  |
| Median age (IQR) | 56 (48-63) |
| Median years since starting to work as general practitioner (IQR) | 18 (8-27) |
| Sex | |
| Women (%) | 737 (36) |
| Men (%) | 1292 (64) |
| Employment status | |
| Independent (%) | 1467 (72) |
| Employed (%) | 564 (28) |
| GP practice type | |
| Group practice (%) | 1288 (66) |
| Single practice (%) | 675 (34) |
| Location | |
| Non-urban (%) | 642 (35) |
| Urban (%) | 1219 (65) |
| Self-dispensation of medications in GP office**^2^** | |
| Yes (%) | 1090 (57) |
| No (%) | 832 (43) |
| Median work percentage (IQR) | 80 (60-100) |
| Type of health records used |  |
| Electronic health records (%) | 430 (72) |
| Paper records (%) | 59 (9) |
| Both (%) | 112 (18) |
| More information about the Workforce-Study: <https://www.mfe-standpunkte.ch/de/ausgabe/ausgabe-22020--43/artikel/hausaerztemangel-aber-mit-licht-am-horizont--76>, accessed November 24, 2020  Abbreviations: GP=general practitioner, IQR=interquartile range | |

**eFigure 1.** FIRE database, FIRE reference cohorts, and OPTICA trial participants.

| **Patient** | **General practitioners (GPs)** |
| --- | --- |
| 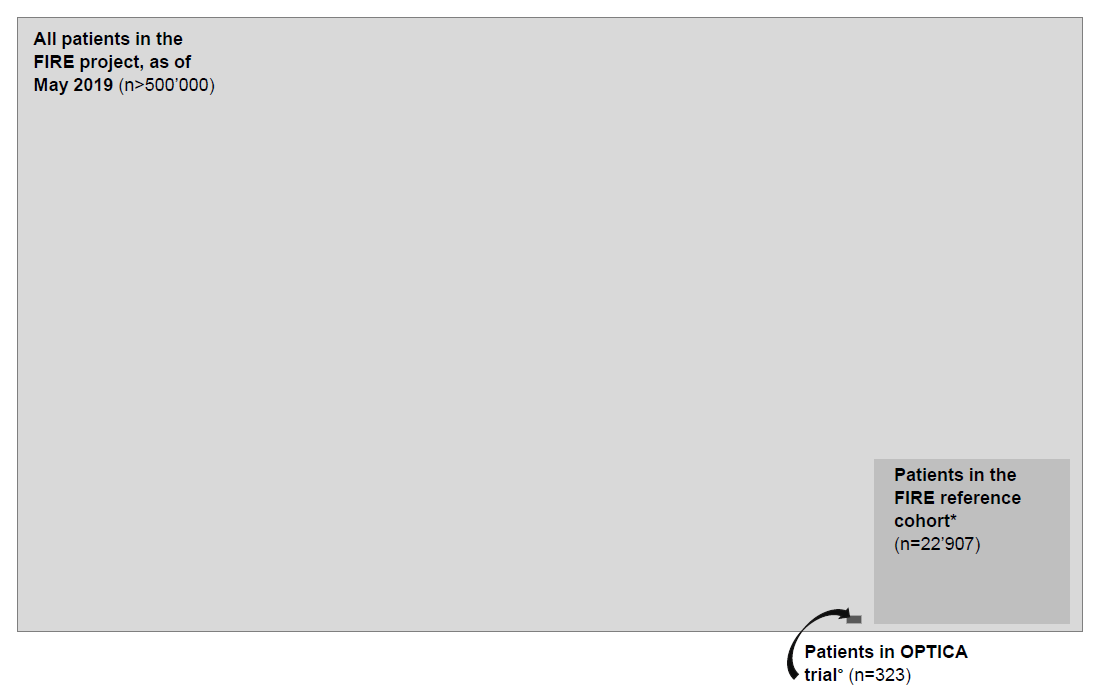 | 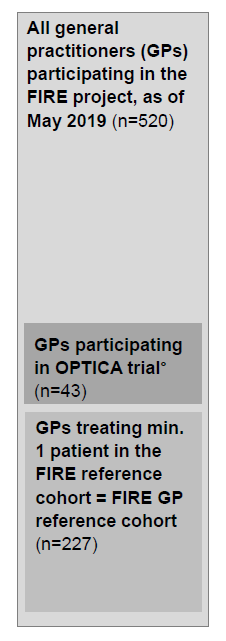 |
| *aged 65 years and over, prescribed at least 5 different medications; °general practitioners who participated in the OPTICA trial also participate in the FIRE project, thus there is information on themselves and their patients in the FIRE database | |

**References**

1. Chmiel C, Bhend H, Senn O, Zoller M, Rosemann T. The FIRE project: a milestone for research in primary care in Switzerland. Swiss Med Wkly. 2011;140:w13142.

2. mfe Haus- und Kinderärzte Schweiz. Hausärztemangel – aber mit Licht am Horizont: Workforce-Studie 2020 – das Wichtigste in Kürze 2020 [Available from: <https://www.mfe-standpunkte.ch/de/ausgabe/ausgabe-22020--43/artikel/hausaerztemangel-aber-mit-licht-am-horizont--76>].

3. Institut für Hausarztmedizin IHAMZ. FIRE-Daten 2019 [Available from: <https://www.hausarztmedizin.uzh.ch/de/fire2/welchedatenbeinhaltetdasprojekt.html>].

4. Jungo KT, Rozsnyai Z, Mantelli S, Floriani C, Löwe AL, Lindemann F, et al. ‘Optimising PharmacoTherapy In the multimorbid elderly in primary CAre’ (OPTICA) to improve medication appropriateness: study protocol of a cluster randomised controlled trial. BMJ open. 2019;9(9):e031080.

5. Shen Z, Meulendijk M, Spruit M. A federated information architecture for multinational clinical trials: STRIPA revisited. 24th European Conference on Information Systems (ECIS 2016); Istanbul, Turkey. 2016.

6. Huibers CJA, Sallevelt BTGM, de Groot DA, Boer MJ, van Campen JPCM, Davids CJ, et al. Conversion of STOPP/START version 2 into coded algorithms for software implementation: A multidisciplinary consensus procedure. International Journal of Medical Informatics. 2019;125:110-7.

7. O'Mahony D, O'Sullivan D, Byrne S, O'Connor MN, Ryan C, Gallagher P. STOPP/START criteria for potentially inappropriate prescribing in older people: version 2. Age and ageing. 2015;44(2):213-8.

8. Hanlon JT, Schmader KE, Ruby CM, Weinberger M. Suboptimal prescribing in older inpatients and outpatients. J Am Geriatr Soc. 2001;49(2):200-9.

9. Hanlon JT, Schmader KE, Samsa GP, Weinberger M, Uttech KM, Lewis IK, et al. A method for assessing drug therapy appropriateness. Journal of clinical epidemiology. 1992;45(10):1045-51.

10. Samsa GP, Hanlon JT, Schmader KE, Weinberger M, Clipp EC, Uttech KM, et al. A summated score for the medication appropriateness index: development and assessment of clinimetric properties including content validity. Journal of clinical epidemiology. 1994;47(8):891-6.

11. Reeve E, Anthony AC, Kouladjian O'Donnell L, Low LF, Ogle SJ, Glendenning JE, et al. Development and pilot testing of the revised Patients' Attitudes Towards Deprescribing questionnaire for people with cognitive impairment. Australasian journal on ageing. 2018;37(4):E150-e4.

12. Reeve E, Low LF, Shakib S, Hilmer SN. Development and Validation of the Revised Patients' Attitudes Towards Deprescribing (rPATD) Questionnaire: Versions for Older Adults and Caregivers. Drugs Aging. 2016;33(12):913-28.

13. Jungo K, Löwe A, Mantelli S, Meier R, Rodondi N, Streit S. Klinische Studie zur Medikamentenoptimierung bei älteren Patient /-innen mit Polypharmazie: Die OPTICA-Studie. Hospital and Primary Care. 2018;18(8):100–2.
